# Cellular and Humoral Immune Responses after Breakthrough Infection in Patients Undergoing Hemodialysis

**DOI:** 10.3390/vaccines11071214

**Published:** 2023-07-06

**Authors:** Masataro Toda, Ayumi Yoshifuji, Tetsuo Nakayama, Setsuko Mise-Omata, Emi Oyama, Yoshifumi Uwamino, Ho Namkoong, Motoaki Komatsu, Akihiko Yoshimura, Naoki Hasegawa, Kan Kikuchi, Munekazu Ryuzaki

**Affiliations:** 1 Department of Nephrology, Tokyo Saiseikai Central Hospital, Tokyo 108-0073, Japan; 2 Department of Infectious Diseases, Keio University School of Medicine, Tokyo 160-8582, Japan; 3 Kitasato Institute for Life Sciences, Laboratory of Viral Infection, Kitasato University, Tokyo 108-8641, Japan; 4 Department of Microbiology and Immunology, Keio University School of Medicine, Tokyo 160-8582, Japan; 5 Department of Laboratory Medicine, Keio University School of Medicine, Tokyo 160-8582, Japan; 6 Division of Nephrology, Shimoochiai Clinic, Tokyo 161-0033, Japan

**Keywords:** COVID-19, booster vaccination, breakthrough infection, hemodialysis

## Abstract

Coronavirus disease 2019 (COVID-19) following primary immunization (breakthrough infection) has been reported in hemodialysis patients; however, their post-infection immune status remains unclear. We evaluated the humoral and cellular immunity of hemodialysis patients after breakthrough infection. Hemodialysis patients who had received primary immunization against COVID-19 at least six months prior to the study but developed mild/moderate COVID-19 before a booster dose (breakthrough infection group) and hemodialysis patients who were not infected with COVID-19 but received a booster dose (booster immunization group) were recruited. In both groups, SARS-CoV-2 antigen-specific cytokines and IgG levels were measured three weeks after infection or three weeks after receiving a booster dose. Memory T and B cells were also counted in the breakthrough infection group using flow cytometry three weeks after infection. Significantly higher SARS-CoV-2 antigen-specific IgG, IFN-γ, IL-5, TNF-α, and IL-6 levels occurred in the breakthrough infection group compared to the booster immunization group (*p* = 0.013, 0.039, 0.024, 0.017, and 0.039, respectively). The SARS-CoV-2 antigen-specific IgG and cytokine levels were not significantly different between the two groups. The breakthrough infection group had significantly higher percentages of central and effector memory T cells and regulatory T cells than the comparison group (*p* = 0.008, 0.031, and 0.026, respectively). Breakthrough infections may induce stronger cellular and humoral immune responses than booster immunizations in hemodialysis patients.

## 1. Introduction

The development of vaccines and their subsequent worldwide implementation against severe acute respiratory syndrome coronavirus 2 (SARS-CoV-2) have been pivotal measures for controlling the global coronavirus disease 2019 (COVID-19) pandemic [1]. The vaccination of hemodialysis (HD) patients was prioritized because of their susceptibility to infection and high mortality rates from COVID-19 [2]. Although recent studies have demonstrated the efficacy of primary vaccines in mitigating COVID-19 infections and severe disease in both the general population and HD patients, HD patients exhibit lower antibody titers than healthy individuals after primary immunization [3]. Consequently, breakthrough infections have been reported in several HD patients after primary immunization, leading to the worldwide recommendation of a third vaccine dose as a booster [4].

To plan appropriate booster immunizations for HD patients with post-breakthrough infections, accurate assessment of the patients’ post-breakthrough immune status is important. Although some studies have demonstrated higher antibody titers following breakthrough infections when compared to those after booster immunization [5], antibody titers in HD patients remain unclear. Moreover, although antibody titers are commonly used to evaluate patients’ immune status against SARS-CoV-2, cellular immunity is crucial, particularly in immunocompromised individuals, such as HD patients. Furthermore, antibody titers may not be strongly linked to COVID-19 prevention in this population [4,6]. Thus, in order to validate HD patients’ immune status against SARS-CoV-2, it is important to assess the entire immune response, including humoral and cellular immunity.

We measured SARS-Cov-2 antigen-specific cytokine production and antibody titers and counted memory T and B cells using flow cytometry to evaluate the immunogenicity of breakthrough infections in HD patients.

## 2. Materials and Methods

This prospective study was approved by the Ethics Committee of the Tokyo Saiseikai Central Hospital (approval number 2021-029).

### 2.1. Participants

The inclusion criteria for HD patients were as follows: patients who had received the primary vaccine series at least six months prior; had not been infected with COVID-19; had not been treated for any malignancy within the past year; had not been treated with steroids, immunosuppressants, or immunomodulators; and had provided written consent for inclusion in this study. The comparison group, who were registered using open recruitment at Tokyo Saiseikai Central Hospital, consisted of patients who fulfilled the conditions set for HD patients, in addition to having an estimated glomerular filtration rate (eGFR) of ≥45 mL/min/1.73 m^2^. All the participants were scheduled to receive a booster dose of the BNT162b2 vaccine. The participants with mild or moderate COVID-19 6–8 months after receiving the primary vaccination but before receiving the booster dose were categorized into the breakthrough infection group. Mild COVID-19 was defined by the presence of various symptoms, including fever and cough, but no shortness of breath or abnormal chest imaging. Moderate COVID-19 was characterized by evidence of lower respiratory tract disease on clinical or imaging evaluation and an oxygen saturation (SpO_2_) of ≥94% in room air at sea level, as measured by pulse oximetry. The participants who were not infected with COVID-19 but received a booster dose were categorized into the booster immunization group.

### 2.2. Antibody Titers

SARS-CoV-2 IgG antibody titers against the S1 subunit of the spike protein of SARS-CoV-2 (anti-S1 antibody titers) were measured using the Abbott SARS-CoV-2 IgG II Quantitative Antibody Assay, which is known to correlate with the neutralizing antibody titers. In the breakthrough infection and booster immunization groups, these titers were measured three weeks after infection or three weeks after receiving a booster dose, respectively. A two-group comparison of anti-S1 antibody titers was performed in all four groups, which included HD patients and controls in both the booster and infected groups.

### 2.3. Cytokine Production Stimulated by the SARS-CoV-2 Spike Protein

A volume of 100 μL of heparinized whole blood was mixed with the same volume of Dulbecco’s Modified Eagle Medium (DMEM) in a 96-well plate coated with 10 ng of spike protein. After 24 h of incubation at 37 °C and 5% CO_2_, the culture supernatant was harvested. The experiment was duplicated for the control culture without stimulation by the spike protein. The culture fluid was stored at −70 °C until the assay was conducted. It was subjected to a cytokine assay using BioPlex human cytokine 17 plex (BIO-RAD Laboratories, Hercules, CA, USA) to examine granulocyte colony-stimulating factor (G-CSF), granulocyte–macrophage (GM)-CSF, interferon (IFN)-γ, interleukin (IL)-1β, IL-2, IL-4, IL-5, IL-6, IL-7, IL-8, IL-10, IL-12, IL-13, IL-17, monocyte chemoattractant protein-1 (MCP-1), macrophage inflammatory protein (MIP)-1β, and tumor necrosis factor (TNF)-α. Net cytokine production was calculated by subtracting the cytokine concentration in the control culture from that in the stimulated culture. A two-group comparison of each cytokine level was performed for all four groups, which included HD patients and controls in both the booster and infected groups.

### 2.4. Flow Cytometry

Blood samples from the breakthrough infection group were obtained two weeks after infection and were diluted with phosphate-buffered saline (PBS). Then, they were gently loaded onto the Lymphoprep^TM^ (Serumwerk Bernburg AG, Bernburg, Germany) layer at a density of 1.007 ± 0.001 g/mL (20 °C), followed by density gradient centrifugation (820× *g*, 25 °C, 30 min). The plasma samples were aliquoted and stored at −20 °C after density gradient centrifugation. The cells were washed with PBS containing 0.5% BSA and 2 mM of EDTA. Then, they were cryopreserved in a CellBanker 1 plus (TAKARA Bio, Kusatsu, Japan) at −80 °C until use. To detect the populations of Tregs, central memory cells, effector memory cells, and circulating follicular T cells, peripheral blood mononuclear cells (PBMC) were stained with monoclonal antibodies against CD3, CD4, CD8, CD25, CD45RA, CCR7, and CD127. To detect anti-RBD IgG antibody-expressing B cells, the thawed PBMCs were incubated with 10 μg/mL of SBP-tagged RBD or 1 μg/mL of biotinylated RBD plus human FcR Blocking Reagent (Militenyi Biotech, Bergisch Gladbach, Germany) in PBS containing 1% FBS, 2 mM of EDTA, and 0.04% NaN_3_ for 30 min at 37 °C. After washing, the cells were stained with a mixture of streptavidin-Brilliant Violet^TM^ (BV)-410 (Biolegend, San Diego, CA, USA) and strepatavidin-PE/Cy7 (Invitrogen, Waltham, MA, USA), monoclonal antibodies against CD20, CD3, CD11b, IgG, CD19, and CD27, and fixable viability dye efluor™ 780 (ThermoFisher Scientific, Waltham, MA, USA). The stained samples were applied to a FACS Canto II (BD, Franklin Lakes, NJ, USA) or Beckman Coulter CytoFlex S (Beckman Coulter, Brea, CA, USA) and analyzed using FlowJo10.5.3 (BD, Franklin Lakes, NJ, USA). The number of anti-RBD IgG antibody-expressing B cells and the percentages of central memory T cells, effector memory T cells, and regulatory T cells were compared between the HD patients and control patients in the breakthrough infection group.

### 2.5. Statistical Analysis

The median values were compared using the Mann–Whitney U test. The frequencies between the groups were compared using Fisher’s exact test or the Chi-square test. Statistical significance was set at *p* < 0.05.

## 3. Results

In total, 29 participants were recruited as controls and 47 participants were recruited as HD patients. Of these, seven participants in the control group and ten participants in the HD group were infected with COVID-19 before receiving the booster dose (the breakthrough infection group). The remaining 22 participants in the control group and 37 participants in the HD group received a booster dose of the BNT162b2 vaccine (the booster immunization group). The characteristics of each group are shown in Table 1.

**Figure 1:**
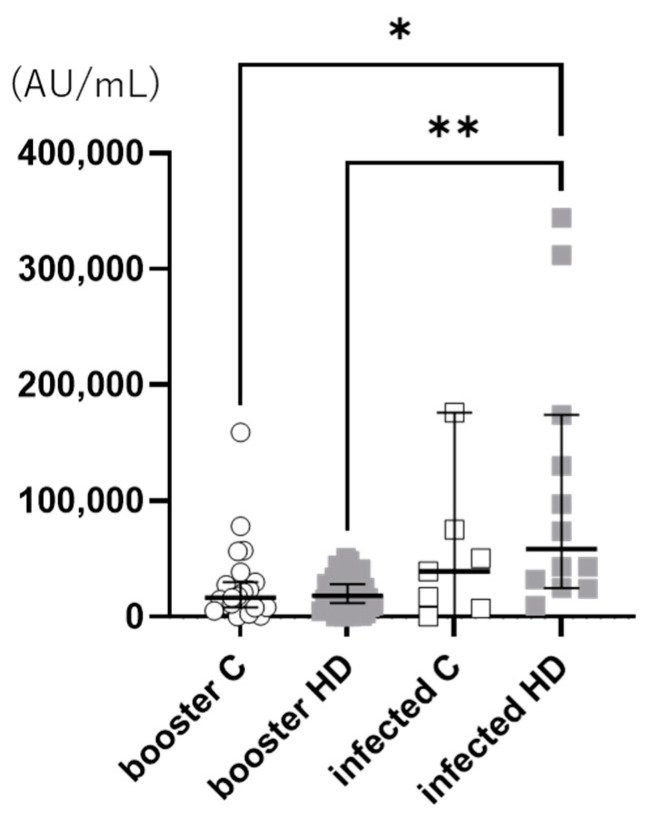
Anti-S1 antibody titers. Anti-S1 antibody titers were measured two weeks after infection in the breakthrough infection group and three weeks after receiving the booster dose in the booster immunization group. HD patients in the breakthrough infection group had a significantly higher anti-S1 antibody titer than those in the booster immunization group (*p* = 0.004). In addition, in the breakthrough infection group, HD patients tended to have higher anti-S1 antibody titers than the controls; however, in the booster immunization group, the anti-S1 antibody titers were similar between the HD patients and the controls. **: *p* ≤ 0.01, *: *p* < 0.05. HD: hemodialysis.

Among the HD patients, the anti-S1 antibody titer in the breakthrough infection group (141,694 ± 76,265 AU/mL) was significantly higher than that in the booster immunization group (29,199 ± 74,998 AU/mL, *p* = 0.004). However, the titers did not differ significantly between the controls in the breakthrough infection and booster immunization groups. In addition, in the breakthrough infection group, HD patients tended to have a higher anti-S1 antibody titer than the controls. Furthermore, in the booster immunization group, the anti-S1 antibody titers were similar between the HD patients and the controls (Figure 1).

**Figure 2:**
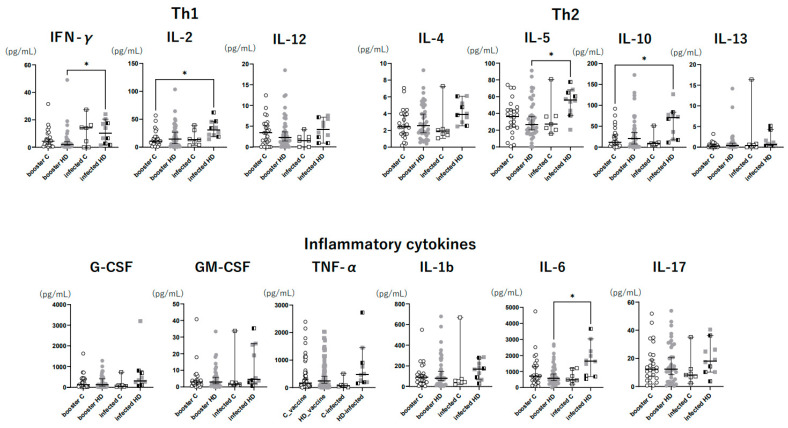
Cytokine levels in response to SARS-CoV-2 antigens. The cytokine levels were measured using SARS-CoV-2 antigen-stimulated blood samples collected two weeks after infection in the breakthrough infection group and three weeks after receiving the booster dose in the booster immunization group. IFN-γ, IL-5, TNF-α, and IL-6 were significantly higher in HD patients after breakthrough infection compared to HD patients who received a booster immunization (*p* = 0.039, 0.037, 0.039, and 0.017, respectively). There was no significant difference in all the cytokine levels between the HD patients and control patients in both the breakthrough infection and booster immunization groups. *: *p* < 0.05. HD: hemodialysis; G-CSF: granulocyte-colony stimulating factor; GM-CSF: granulocyte–macrophage colony-stimulating factor; IFN: interferon; IL: interleukin; TNF-α: tissue necrotic factor-α.

Regarding the Th1-type cytokines, including IFN-γ, IL-2, and IL-12, the HD patients in the breakthrough infection group had higher IFN-γ levels than those in the booster immunization group (*p* = 0.039), but there was no significant difference between the control and HD patients in either of the groups. Regarding the Th2-type cytokines, including IL-4, IL-5, IL-10, and IL-13, the HD patients in the breakthrough infection group had higher IL-5 levels than those in the booster immunization group (*p* = 0.024), but there was no significant difference between the control and HD patients in either group. Regarding the inflammatory cytokines, including G-CSF, GM-CSF, TNF-α, IL-1b, IL-6, and IL-17, the HD patients in the breakthrough infection group had higher TNF-α and IL-6 levels than those in the booster immunization group (*p* = 0.017 and 0.039, respectively). However, there was no significant difference between the control and HD patients in either group (Figure 2).

**Figure 3:**
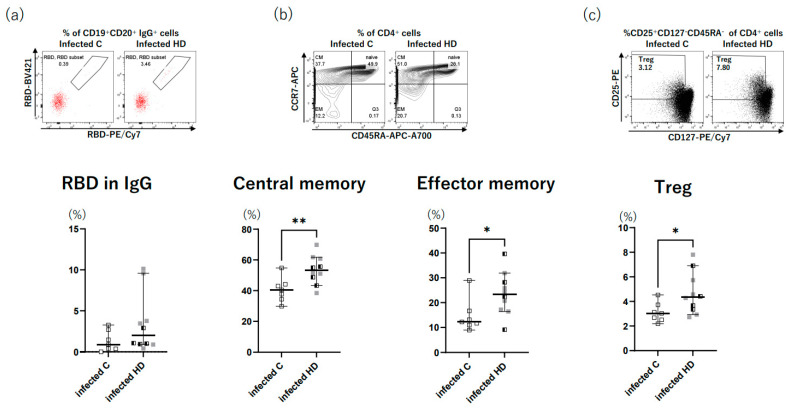
The analysis of memory T and B cells. Peripheral blood mononuclear cells were sampled two weeks after breakthrough infection and were stained with monoclonal antibodies, and memory T cells and anti-RBD IgG antibody-expressing B cells were counted using flow cytometry. (**a**) The number of anti-RBD IgG antibody-expressing B cells tended to be higher in HD patients than in control patients. (**b**) The percentage of central memory T cells was significantly higher in HD patients than in control patients (*p* = 0.008). The percentage of effector memory T cells was significantly higher in HD patients than in control patients (*p* = 0.031). (**c**) The percentage of regulatory T cells was significantly higher in HD patients than in control patients (*p* = 0.026). The representative fluorescence-activated cell sorting (FACS) profiles for control and HD patients are shown above each figure (**a**–**c**). **: *p* ≤ 0.01; *: *p* < 0.05. HD: hemodialysis.

The flow cytometry results for the breakthrough infection group are shown in Figure 3. The HD patients tended to have a larger number of anti-RBD IgG antibody-expressing B cells than the control group (Figure 3a). In addition, the HD patients had a significantly higher percentage of central memory T cells, effector memory T cells, and regulatory T cells than the control group (*p* = 0.008, 0.031, and 0.026) (Figure 3b,c).

## 4. Discussion

We conducted a comparative analysis of the immune responses of HD patients and control patients after booster immunization and breakthrough infection. Our study revealed that HD patients acquired significantly higher levels of SARS-CoV-2 IgG and SARS-CoV-2-specific cytokines following breakthrough infection compared to following booster immunization. In addition, the HD patients tended to have higher humoral immune responses, such as memory B cells and cellular immune responses, after breakthrough infection than the control patients.

The antibody titer against SARS-CoV-2 decreases gradually over time after receiving the primary vaccine series, and the booster dose effectively increases the antibody titer. Although the antibody titers in HD patients after receiving the primary series were lower than those in the general population, the antibody titers in HD patients were not lower and were even higher than those in the general population after receiving a booster dose in some reports [7]. In the present study, there was no significant difference in antibody titers between the HD patients and the control patients after receiving a booster dose.

The risk of breakthrough infections is high six months after primary immunization, when antibody titers wane [8]. After breakthrough infections, the antibody response is typically boosted within seven days of symptom onset or polymerase chain reaction (PCR) test positivity [9]. Antibody titers in patients with moderate to severe breakthrough infections are higher than those in asymptomatic or mild cases, regardless of the SARS-CoV-2 variant strains causing the infection [8,9]. In the present study, the anti-S1 antibody titer in the breakthrough infection group was significantly higher than that in the booster immunization group among the HD patients; however, they were similar in the control group. In addition, the HD patients in the in the breakthrough infection group tended to have higher anti-S1 antibody titers and more anti-RBD IgG antibody-expressing B cells than those in the control group. The reason why the HD patients acquired a strong humoral response through breakthrough infection may be that HD patients have impaired immune defenses compared to control patients [10]. Similar to the HD patients in this study, kidney transplant recipients have significantly higher serologic responses after infection compared to after vaccination [11], whereas in the general population, the serologic responses after infection and after vaccination are similar [5]. Furthermore, kidney transplant recipients with mild COVID-19 infection had a stronger serologic response compared to healthy controls [11]. Furthermore, the negative correlation between age and antibody titers is usually observed after vaccination due to decreased immune function associated with old age, but this was not observed after breakthrough infection [5]. This is because immunocompromised patients, who cannot fight the early viral invasion due to a lack of effective immune defenses, mount a higher viral load. Therefore, they mount a higher serologic response [11,12]. Similarly, HD patients who are immunocompromised cannot adequately reduce their viral loads, which may activate a stronger vaccine-acquired memory immune response against SARS-CoV-2. No studies have been reported on whether the infected group has a stronger response than the vaccinated group in hemodialysis patients for other infections. However, in pediatric populations, in which immune responses have not been established, the influenza vaccination group has been reported to have a lower immune response than the infected group [13]. This result suggests that the naturally infected group could provoke a stronger immune response for other infections compared to the vaccinated group in patients with impaired immune function.

We also evaluated cellular immune responses by measuring the levels of various cytokines in response to SARS-CoV-2 antigens. This method involved stimulation with spike protein, which reacts to epitopes other than RBDs. This method enabled us to measure Th1, Th2, and inflammatory cytokines other than IFN-γ, as Brueggeman et al. and Yamaguchi et al. reported [14,15]. Our findings revealed that inflammatory cytokines, including TNF-α and IL-6, Th1-related cytokine IFN-γ, and Th2-related cytokine IL-5 were significantly elevated in HD patients after breakthrough infection compared to after booster immunization. IL-6 is a pleiotropic cytokine that exerts both pro- and anti-inflammatory effects in response to tissue damage caused by viral infections. IL-6 acts as a key mediator of cytokine storms caused by COVID-19; therefore, tocilizumab is used in severe cases of COVID-19 [16,17]. IL-6 activates cytotoxic T lymphocytes and then stimulates the release of IFN-γ [18]. Additionally, IL-6 stimulates CD4+ T cells to secrete IL-4, thereby inducing a Th2 response that promotes antibody production [19]. In HD patients, the breakthrough infection induced a stronger cellular immune response than the booster dose, as evidenced by the cytokine responses.

Furthermore, although not COVID-19-specific, we examined memory T cells in the breakthrough infection group and found that the HD patients had significantly higher central and effector memories than the control group. Upon antigen stimulation, naïve T cells differentiate into long-lived central memory T cells and short-lived effector T cells [20]. Although the presence of moderate COVID-19 cases in HD patients is an important issue to consider, memory immunity may be more strongly induced in HD patients compared to control patients. Figure 2 shows that the levels of inflammatory cytokines, including TNF-α, IL-1b, and IL-6, tended to be higher in infected HD patients than in infected control patients. Impaired innate immunity in HD patients may result in higher cytokine production against SARS-CoV-2 and induce a greater memory T cell response. Regulatory T cells, which are responsible for counteracting the overactivation of the immune response by producing IL-10, decrease with increasing HD vintage in HD patients [21]. However, a previous study demonstrated that COVID-19-infected HD patients have higher numbers of CD39+ regulatory T cells than COVID-19-infected control patients [22]. In the present study, HD patients had a significantly higher percentage of regulatory T cells and tended to have higher IL-10 levels compared to control patients after breakthrough infection. A strong cytokine response against breakthrough infection may induce regulatory T cells in HD patients, more so than in control patients.

As a limitation, we could not identify the SARS-CoV-2 strain in patients with breakthrough infections. In addition, we could not equalize the number of cases in terms of the severity of COVID-19 between the HD patients and controls in the breakthrough infection group. Furthermore, COVID-19-specific memory T cells were not analyzed because an insufficient blood sample volume was collected.

## 5. Conclusions

HD patients acquired significantly higher levels of SARS-CoV-2 IgG and SARS-CoV-2-specific cytokines following breakthrough infection compared to booster immunization. These findings suggest that breakthrough infections may induce stronger cellular and humoral immune responses than booster immunizations in HD patients.

## Figures and Tables

**Table 1:** Background characteristics.

		Breakthrough Infection		Booster Immunization	
		Control (n = 7)	HD (n = 10)	Control (n = 22)	HD (n = 37)
Age (years)		58.9 ± 14.2	69.5 ± 11.4	70.5 ± 8.8	78.7 ± 9.0
Males (n, (%))		7 (77.7)	10 (90.9)	17 (77.3)	22 (59.5)
BMI		25.3 ± 3.9	23.6 ± 5.4	23.5 ± 3.4	22.2 ± 3.5
Diabetes mellitus (n, (%))		6 (85.7)	8 (80.0)	4 (18.2)	15 (40.5)
Dyslipidemia (n, (%))		2 (27.6)	4 (40.0)	5 (22.7)	16 (43.2)
Malignant tumor (n, (%))		0	2 (20.0)	0	1 (2.7)
Cardiovascular disease (n, (%))		0	5 (50.0)	1 (4.5)	11 (29.7)
Cerebrovascular disease (n, (%))		1 (14.3)	2 (20.0)	1 (4.5)	6 (16.2)
COVID-19	Mild	7	4		
	Moderate	0	6		

HD, hemodialysis; BMI, Body Mass Index; COVID-19, coronavirus disease 2019.

## Data Availability

The datasets generated and analyzed in this study are available from the corresponding author upon reasonable request.
